# Redox conduction facilitates direct interspecies electron transport in anaerobic methanotrophic consortia

**DOI:** 10.1126/sciadv.adw4289

**Published:** 2025-08-22

**Authors:** Hang Yu, Shuai Xu, Yamini Jangir, Gunter Wegener, Victoria J. Orphan, Mohamed Y. El-Naggar

**Affiliations:** ^1^ College of Urban and Environmental Sciences, Peking University, Beijing 100871, China.; ^2^ Department of Physics and Astronomy, University of Southern California, Los Angeles, CA 90089, USA.; ^3^ Division of Geological and Planetary Sciences, California Institute of Technology, Pasadena, CA 91125, USA.; ^4^ MARUM, Center for Marine Environmental Sciences, University of Bremen, Bremen 28359, Germany.; ^5^ Max Planck Institute for Marine Microbiology, Bremen 28359, Germany.

## Abstract

Anaerobic methanotrophic archaea (ANME) and sulfate-reducing bacteria (SRB) form syntrophic partnerships in marine sediments to consume greenhouse gas methane. While direct interspecies electron transport is proposed to enable ANME/SRB symbiosis, its electrochemical properties remain uncharacterized. Here, using sediment-free enrichment cultures, we measured the electron transport capabilities of marine consortia under physiological conditions. Diverse ANME/SRB consortia exhibited high dry conductance close to electrogenic biofilms. This conductance diminished upon exposure to heat or oxygen but was preserved following paraformaldehyde fixation, indicating a biomolecular origin for this electric charge transfer. Cyclic voltammetry revealed redox activity centered at 28 ± 11, 94 ± 6, and 24 ± 7 millivolts for ANME-1/*Desulfofervidus*, ANME-2a/Seep-SRB1, and ANME-2a+2c/Seep-SRB1+2 consortia, respectively. Generator-collector measurements further demonstrated that these redox components facilitate electron transport over micrometer-scale distances, sufficient to link archaeal and bacterial partners. Collectively, our results establish that marine ANME/SRB symbiosis uses redox conduction, consistent with multiheme cytochrome *c*, for direct interspecies electron transport.

## Teaser

Methane-consuming microbial partners use redox conductors to pass electrons.

## INTRODUCTION

An increasing number of microorganisms have been demonstrated or hypothesized to perform extracellular electron transfer, which is the ability to move electrons across their cell membranes to or from external materials or surfaces (*1*, *2*). This ability allows microorganisms to react with insoluble extracellular substrates (*3*–*9*) and to form artificial cocultures (*10*, *11*).

A natural microbial symbiosis catalyzes the anaerobic oxidation of methane in marine ecosystems worldwide (*12*, *13*). Anaerobic methane oxidation coupled to sulfate reduction is a major sink of greenhouse gas methane (*14*) and is mediated by consortia of anaerobic methanotrophic archaea (ANME) and sulfate-reducing bacteria (SRB). Recent studies suggest that their syntrophic interaction is facilitated by direct interspecies electron transport, in which methane-derived electrons are directly conducted from ANME to SRB to drive sulfate reduction (*15*–*20*). Two different mechanisms may enable direct interspecies electron transport in this marine microbial syntrophy: metallic conduction using conductive pili (*16*, *21*) or amorphous carbon (*22*) or redox conduction using redox-active molecules (*23*) or multiheme cytochrome *c* (*15*, *21*, *24*, *25*). While ANME/SRB consortia thrive in sulfate-rich marine sediments, a distinct family of ANME, *Candidatus* Methanoperedens (ANME-2d), has been identified in freshwater environments, where they couple methane oxidation to electron acceptors such as nitrate, iron, and manganese (*26*–*29*). Recent electrochemical studies of *Ca.* Methanoperedens enrichments demonstrated that these archaea use multiheme cytochrome *c* to transport electrons to metals and electrodes (*30*, *31*), raising the possibility that similar electron transport mechanisms may be at play in marine ANME/SRB syntrophy.

In this study, we performed direct solid-state (dry) conductance and electrochemistry (in solution) measurements on ANME/SRB consortia to shed light on possible electrical connections in these syntrophic cellular aggregates. Electrochemical analyses are very sensitive to sample impurities. However, ANME/SRB consortia naturally reside in marine sediments. Both sediment particles and the presence of other diverse microbial taxa could interfere and complicate the interpretations of the measurement results. We circumvented this issue using long-term sediment-free enrichment cultures of ANME/SRB consortia from different marine methane seep environments including cold seeps, clastic sediments, and hot seeps (*16*, *32*, *33*). Planktonic cells and electrogenic biofilms of the model extracellular electron transfer organism *Geobacter sulfurreducens* were used as controls. We present the first electrical and electrochemical measurements of three diverse ANME/SRB partnerships, representing distinct phylogenetic families and orders of archaea and bacteria, to establish redox conduction as the fundamental syntrophic mechanism in these key microbial consortia involved in global methane cycling.

## RESULTS

### Electrical conductance of ANME/SRB consortia

**Fig. 1.**
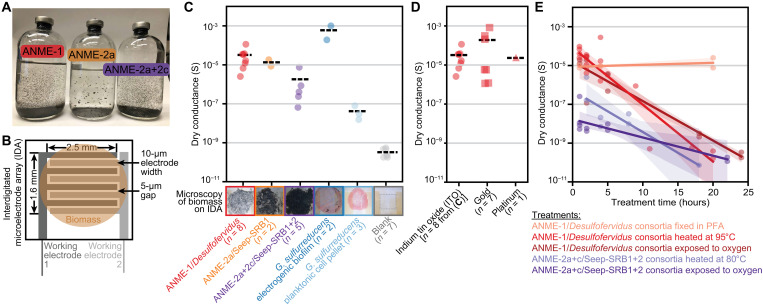
Dry conduction measurements of ANME/SRB consortia and *G. sulfurreducens*. (**A**) Sediment-free enrichments of ANME-1/*Desulfofervidus*, ANME-2a/Seep-SRB1, and ANME-2a+2c/Seep-SRB1+2 consortia (left to right). (**B**) Schematic of the interdigitated microelectrode array (IDA), consisting of two 10-μm-wide working electrodes separated by a 5-μm gap. Biomass was dried onto the IDA to promote physical contact with the electrode surface. Conductance was determined from the linear slope of the current-voltage relationship measured across the two working electrodes. (**C**) ANME-1/*Desulfofervidus*, ANME-2a/Seep-SRB1, and ANME-2a+2c/Seep-SRB1+2 consortia and *G. sulfurreducens* electrogenic biofilm show high conductance when dried on the indium tin oxide (ITO) IDAs, whereas *G. sulfurreducens* nonelectrogenic planktonic cell pellets show low conductance. Black dashed lines indicate the means of the data points. Representative light microscopy of IDAs with dried biomass is shown above the sample names; see fig. S7 for enlarged microscopy images. Dashed line area in the blank indicates the working electrode area of an ITO IDA. The number of biological replicates is included in brackets after the sample names. (**D**) Dry conduction measurements of ANME-1/*Desulfofervidus* consortia on different IDA materials show comparable conductances. Black dashed lines indicate the means of the data points. The number of biological replicates is included in brackets after the material types. (**E**) Dry conduction of ANME-1/*Desulfofervidus* and ANME-2a+2c/Seep-SRB1+2 consortia remains the same with paraformaldehyde (PFA) fixation but decreases with heating and oxygen exposure. Solid lines represent linear regressions of the plotted data, and shaded areas represent the 95% confidence intervals of the linear regressions.

Multiyear enrichment cultivation resulted in sediment-free enrichment cultures of ANME/SRB consortia, composed mainly of three different ANME lineages with unique syntrophic SRB partners (Fig. 1A and fig. S1) (*16*, *32*, *33*). These include ANME-1 partnered with sulfate-reducing *Desulfofervidus* (ANME-1/*Desulfofervidus* consortia) grown at 50°C, ANME-2a partnered with Seep-SRB1 (ANME-2a/Seep-SRB1 consortia) grown at 10°C, and ANME-2a and ANME-2c partnered with Seep-SRB1 and Seep-SRB2 grown at 20°C (ANME-2a+2c/Seep-SRB1+2 consortia). The 16*S* ribosomal RNA (rRNA) gene amplicon sequencing showed ANME as the most abundant species varying from 25 to 57% of the total communities and did not detect known electroactive bacteria such as *Geobacter* and *Shewanella* (fig. S1). The ANME/SRB consortia have slow growth rates with doubling times of 1 to 7 months, yielding limited biomass for experiments even after several years of cultivation (*16*, *32*, *33*). Similar to methane seep sediments, ANME and SRB in these sediment-free enrichment cultures grow in close proximity to each other, forming many-celled consortia with diameters of tens to hundreds of micrometers, and both ANME and SRB cells can be exposed at the surface of the aggregates (*16*, *32*, *33*). In these cultures, ANME/SRB consortia further aggregate into larger flocs, >100 μm in size, that are visible to the naked eye (Fig. 1A). This is in contrast to methane seep sediments, in which in situ ANME/SRB consortia tend to be small and attached to sediment particles (*32*), complicating direct measurements of their electrochemical properties.

We first characterized the dry conductance of the different ANME/SRB consortia using the well-studied model electroactive bacteria *G. sulfurreducens* as controls. Because of the limited biomass availability of ANME/SRB consortia, we performed solid-state measurements using interdigitated microelectrode arrays (IDAs), which required only 0.1 ml of concentrated biomass per experiment. Within an area of 2.6 mm by 1.6 mm, the arrays on the IDA contained 65 pairs of 10-μm-wide microelectrodes separated by a 5-μm gap (Fig. 1B). This electrode array pattern had previously been used to investigate micrometer-scale electron transport in electroactive bacteria such as *Geobacter*, *Shewanella*, and *Pseudomonas* by growing cells on the microelectrodes (*34*–*37*). Given the long generation times of ANME/SRB consortia, we test directly depositing samples onto the IDA after briefly washing with deionized water to remove salts in the media that could possibly contribute to the measured conduction. To promote physical attachment between cells and the electrodes, we dried the washed and concentrated biomass onto the IDAs. Control measurements of *G. sulfurreducens* showed different dry conductance depending on the cell growth condition: Cell pellets centrifuged from fumarate-respiring planktonic cells (nonelectrogenic condition) showed a dry conductance of 4.2 × 10^−8^ S (*n* = 2), whereas electrogenic biofilms peeled from graphite electrodes (electrogenic condition) showed four orders of magnitude higher conductance of 6.0 × 10^−4^ S (*n* = 3) (Fig. 1C). This difference in *G. sulfurreducens* conductance could be explained by cells primed for extracellular electron transfer when grown as an electrogenic biofilm (*38*), although our measurements were performed on peeled electrogenic biofilms.

ANME/SRB consortia showed high dry conductance sensitive to elevated temperature and oxygen. The three tested ANME/SRB consortia produced electric conductance with means of 3.2 × 10^−5^ S (*n* = 8), 1.4 × 10^−5^ S (*n* = 2), and 0.2 × 10^−5^ S (*n* = 5) for ANME-1/*Desulfofervidus*, ANME-2a/Seep-SRB1, and ANME-2a+2c/Seep-SRB1+2 consortia, respectively (Fig. 1C and fig. S2). The means of dry conductance observed for ANME/SRB consortia were two orders of magnitude higher than those of nonelectrogenic fumarate-respiring *G. sulfurreducens* cells, with values an order of magnitude lower than those of electrode-respiring *G. sulfurreducens* biofilms (Fig. 1C). Our reported conductance values allow for relative comparisons within our study, with the caveat that they are not conductivity values, which account for the thickness of biomass deposited on the IDAs, and the variability observed among biological replicates may partly reflect this difference. These dry conductances of the ANME/SRB consortia were similar on different electrode materials, including gold, platinum, and indium tin oxide (ITO); thus, we opted to use ITO for subsequent measurements (Fig. 1D). Moreover, to distinguish conduction by abiotic materials such as amorphous carbon (*22*) or redox species (*23*) from that by biologically derived protein structures such as conductive pili (*16*, *21*) or multiheme cytochrome *c* (*16*, *21*, *24*, *25*), we subjected dried ANME/SRB consortia on IDAs to treatments with different effects on biomolecular integrity. Specifically, we applied elevated temperature and oxygen exposure to denature proteins but unlikely to affect chemical or mineral conductors, as well as formaldehyde fixation to wash away soluble redox species while cross-linking and preserving protein structures. The dry conductance of the anaerobic consortia was lost within a day following heat or oxygen exposure but was retained in biomass fixed and washed with anoxic paraformaldehyde (Fig. 1E), indicating that a direct conduction path via proteins is likely responsible for the observed high conductance. On the basis of the consistently high dry conductance across diverse ANME/SRB consortia and their sensitivity to treatments affecting protein integrity, we next investigated the electrochemical properties of ANME/SRB consortia under physiological conditions.

### Redox properties of ANME/SRB consortia

**Fig. 2.**
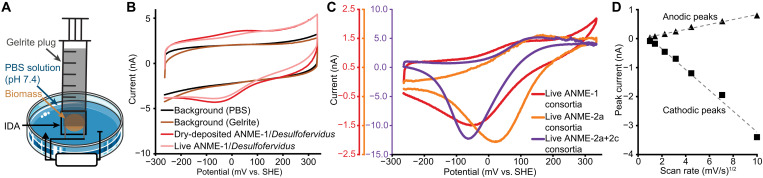
Cyclic voltammetry measurements of dried and live ANME/SRB consortia. (**A**) Live biomass was pressed onto the IDA using a Gelrite plug in a syringe to promote physical attachment and electrochemistry measurements under anoxic conditions in PBS solution (pH 7.4). A platinum wire served as the counter electrode and Ag/AgCl (1 M KCl) as the reference electrode. (**B**) Representative cyclic voltammograms of dry-deposited and live ANME-1/*Desulfofervidus* consortia show clear redox signals, whereas PBS and Gelrite backgrounds show no redox signal. (**C**) Representative cyclic voltammograms show redox peaks in diverse ANME/SRB consortia after subtraction of PBS background current from the same IDAs. (**D**) Comparison of peak current heights for ANME-1/*Desulfofervidus* consortia subjected to increasing scan rates, revealing a linear dependence on the square root of scan rates and indicating that electron transport through the consortia is rate limiting.

We investigated the redox activity via cyclic voltammetry in a standard three-electrode electrochemical setup (Fig. 2A). In cyclic voltammetry techniques, the electrical current is monitored as a function of electrical potential, and the rate of potential change can yield insight into the redox and charge transfer properties of a sample on the electrode. The millimolar concentrations of sulfide produced by ANME/SRB consortia can mask the electrochemical detection of soluble redox-active molecules present at lower concentrations (for example, thioquinoxalinol sulfate reported from ANME-1/*Desulfofervidus* enrichments) (*23*). However, our dry conduction measurements of ANME/SRB biomass point to a direct conduction path rather than conduction via soluble redox-active compounds. For our live conduction measurements, we washed concentrated ANME/SRB consortia at least six times using anoxic phosphate-buffered saline (PBS; pH 7.4) solution to eliminate sulfide and any other soluble molecules in the samples before analysis. We note that redox-active molecules bound to proteins, as is the case for flavin and 2-amino-3-carboxy-1,4-naphthoquinone in *Shewanella oneidensis* (*39*–*41*), would still be detected. To adhere the consortia to the electrode surface, we either dried consortia biomass onto the IDAs as the dry conduction measurements (dry-deposited) or directly pressed alternatively concentrated consortia onto the IDAs using a Gelrite plug without drying (live) (Fig. 2A). We confirmed the effectiveness of this live anaerobic measurement approach by pressing nonelectrogenic cell pellets and electrogenic biofilms of *G. sulfurreducens* onto the IDAs (fig. S3). Notably, both the dried and live *G. sulfurreducens* showed similar redox couples that matched multiheme cytochrome *c* reported previously (*38*). Specifically, we observed that nonelectrogenic fumarate-respiring cells exhibited multiple redox components, whereas the electrogenic biofilms showed only a single redox component [midpoint potential *E*_1/2_ = 34 ± 19 mV versus standard hydrogen electrode (SHE), *n* = 5; fig. S3] that corresponds to the most positive potential for cytochrome *c* reported in the previous study (*38*). Overall, this live anaerobic cyclic voltammetry approach could be applied to other electroactive microorganisms for measuring redox components, such as multiheme cytochrome *c* (*15*, *21*, *24*).

Both dry-deposited and live ANME-1/*Desulfofervidus* consortia in PBS solution (pH 7.4) showed a robust redox couple (*E*_1/2_ = 28 ± 11 mV versus SHE, *n* = 5; Fig. 2B and fig. S4). The cathodic peak currents (*E*_cathodic_ = −53 mV) were consistently higher than the anodic peak currents (*E*_anodic_ = +109 mV), similar to our observations and previous reports for electrode-respiring *G. sulfurreducens* (*36*, *42*) (fig. S3). These redox signals detected are not from the PBS solution, Gelrite plug, or sulfide. Control measurements of the PBS solution and Gelrite plugs did not show any redox properties (Fig. 2B), and a control measurement of the ANME-1/*Desulfofervidus* consortia containing sulfide displayed an additional cathodic peak at −275 mV that diminished over successive scan cycles and lacked a corresponding anodic peak (fig. S5). This sulfide signal was not observed in washed samples, supporting that the wash steps effectively removed sulfide or other soluble molecules before electrochemical analysis.

Analysis of the ANME-2a/Seep-SRB1 consortia revealed a redox component similar to that of the ANME-1/*Desulfofervidus* consortia (*E*_1/2_ = 94 ± 6 mV versus SHE, *E*_cathodic_ = −3 mV, *E*_anodic_ = 185 mV versus SHE, *n* = 3), and a similar component was also observed in ANME-2a+2c/Seep-SRB1+2 consortia (*E*_1/2_ = 24 ± 7 mV versus SHE, *E*_cathodic_ = −65 mV, *E*_anodic_ = 113 mV versus SHE, *n* = 3) (Fig. 2C). This single redox component observed across diverse marine ANME/SRB consortia is in line with recently reported measurements of the freshwater methanotrophic *Ca.* Methanoperedens by Zhang and colleagues (*31*) (*E*_1/2_ = −20 mV) and one of the two redox components (*E*_1/2_ = 100 mV but not *E*_1/2_ = −180 mV) observed by Ouboter and colleagues (*30*). In cyclic voltammetry measurements of dried ANME-1 consortia, the redox component disappeared upon heating and oxygen exposure, but not with anoxic paraformaldehyde fixation (fig. S6), similar to the disappearance of dry conduction observed in these same consortia (Fig. 1E). Furthermore, the redox component’s anodic and cathodic peak currents were both proportional to the square root of scan rates (Fig. 2D), indicating an apparent electron diffusion behavior in which the homogeneous electron transport within the ANME/SRB consortia was slow compared to the heterogeneous electron transport from ANME/SRB consortia to the IDA. This diffusional behavior has been previously observed in electrode-respiring *Geobacter* biofilms, where cytochrome-mediated electron transport through the biofilm is rate limiting (*36*, *38*, *43*). Similarly, the redox component measured here, possibly multiheme cytochrome *c* (*15*, *24*), in diverse syntrophic lineages of ANME/SRB consortia may also be rate limiting for direct interspecies electron transport.

### Redox conduction in ANME/SRB consortia

**Fig. 3.**
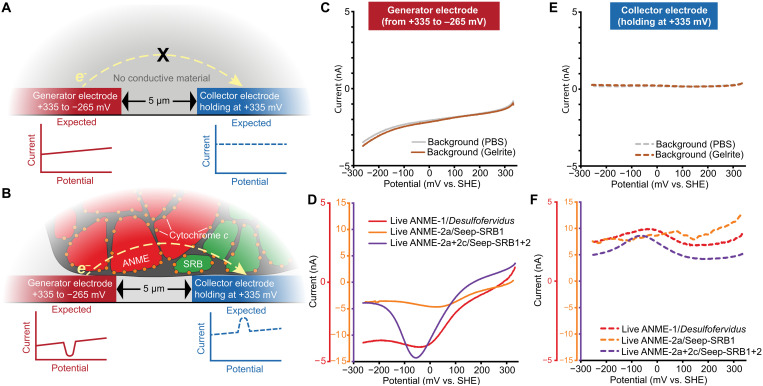
Generator-collector measurements of ANME/SRB consortia. (**A** and **B**) Schematics of expected outcomes for generator-collector measurements: (A) a blank background lacking conductive material and (B) ANME/SRB consortia containing redox conductive cytochromes that mediate electron transport across the 5-μm gap. The generator electrode sweeps to a more negative potential, reducing the redox component, while the collector electrode is held at a constant positive potential, reoxidizing it. (**C** and **D**) Current measured at the generator electrode sweeping from +335 to −265 mV for (C) backgrounds and (D) live ANME/SRB consortia. (**E** and **F**) Currents measured at the collector electrodes holding at a constant +335 mV for (E) backgrounds and (F) live ANME/SRB consortia. Together, the generator-collector measurements reveal that the redox-active components, consistent with multiheme cytochrome *c*, in diverse ANME/SRB consortia are capable of facilitating micrometer-scale electron transport from generator electrodes to collector electrodes.

To test whether the redox-active component observed in ANME/SRB consortia could mediate long-distance electron transport across multiple cells, we measured electron conduction across the gap between the two IDA working electrodes using the generator-collector method (*37*). In this four-electrode setup, two IDA working electrodes shared the same reference and counter electrodes (Fig. 3A). A voltage difference between the two working electrodes was used to drive electron transport across the 5-μm gap. One working electrode served as the “generator” of electrons by sweeping from a positive potential to a more negative potential, while the other working electrode served as the “collector” of electrons by poising at a fixed positive potential. The generation of current would only be detected at the collector electrode in the presence of a conductive path, which allowed a migration of electrons from the more negative generator to the more positive collector electrode (Fig. 3A). If electron transport was facilitated by a redox component, then the current at the collector electrode should be restricted to the specific redox potential of that component (Fig. 3B). Anoxic generator-collector control measurements of pure PBS and Gelrite showed that the generator electrode reflects a charging behavior without a redox signal (Fig. 3C), and, as expected in the case without a conductive path, current at the collector electrode remained stable (Fig. 3E). In anoxic generator-collector measurements of all three lineages of live ANME/SRB consortia, a current peak was observed in the generator electrode only at the potential matching that of the redox component observed in cyclic voltammetry measurements (Figs. 2D and 3D), indicating that electrons left the generator electrode. A corresponding current peak of similar shape and potential but opposite sign and lower magnitude indicated that electrons from the generator electrode moved to the collector electrode facilitated by the redox component (Fig. 3F). This dependency of collector current on the generator electric potential is evidence that the reduction of the redox component at the generator electrode and the oxidation of the same redox component at the collector electrode facilitate electron transport in ANME/SRB consortia across the 5-μm gap. The results are consistent with electron transport via multiheme cytochrome *c* in *Geobacter* reported previously and performed here (fig. S3). Together, these results indicate that the redox components present in diverse ANME/SRB consortia are capable of transporting electrons over several micrometers, enough to connect methane oxidation in ANME to sulfate reduction in their SRB partners.

## DISCUSSION

An increasing number of microorganisms have been hypothesized or shown to be electroactive (*1*, *44*). Since the discovery of ANME/SRB consortia 25 years ago (*12*, *13*, *45*), the mechanism of this archaeal-bacterial symbiosis has been intensively studied (*14*, *46*). Direct interspecies electron transport has emerged as the most likely syntrophic mechanism linking ANME to their SRB partners (*15*–*17*, *21*, *24*, *47*, *48*); however, the electrical and electrochemical properties of these consortia have not been directly measured to reveal the underlying electron transport mechanism. We evaluated two previously proposed electron transport mechanisms in ANME/SRB consortia: metallic conduction via conductive pili (*16*, *21*) or amorphous carbon (*22*) and redox conduction via redox-active molecules (*23*) or multiheme cytochrome *c* (*16*, *21*, *24*, *25*). Three different ANME/SRB consortia exhibit high conductance close to electrogenic *Geobacter* biofilms (Fig. 1). This dry conductance is sensitive to heat and oxygen but not paraformaldehyde fixation and washes, indicative of protein involvement. The electrochemical characteristics are inconsistent with metallic conduction via pili (*16*), which would show linear current-potential relationships. Instead, we show distinct redox peaks capable of long-distance electron transport, consistent with a redox conduction mechanism (Figs. 2 and 3).

On the basis of genomic and transcriptomic results, the most likely redox conductor in ANME/SRB consortia is multiheme cytochrome *c*. These have been predicted to localize in the membrane and span the archaeal S-layer to transport electrons out of ANME (*15*, *21*, *24*, *48*) and into SRB cells (*21*, *25*, *49*). Electron microscopy has also observed cytochromes and nanowire-like structures in the intracellular space (*15*, *21*), which could form a network to facilitate electron transport over micrometer-scale distances, as observed in our assays (Fig. 3). While we cannot fully exclude contributions from redox-active molecules such as thioquinoxalinol (*23*), this compound has so far only been identified in ANME-1/*Desulfofervidus* consortia. In addition, our dry conduction measurements of ANME/SRB biomass suggest a direct electron conduction pathway rather than transport mediated by soluble redox-active molecules (Fig. 1). Thus, multiheme cytochrome *c* is more likely the primary redox-active component facilitating redox conduction in ANME/SRB consortia.

**Fig. 4.**
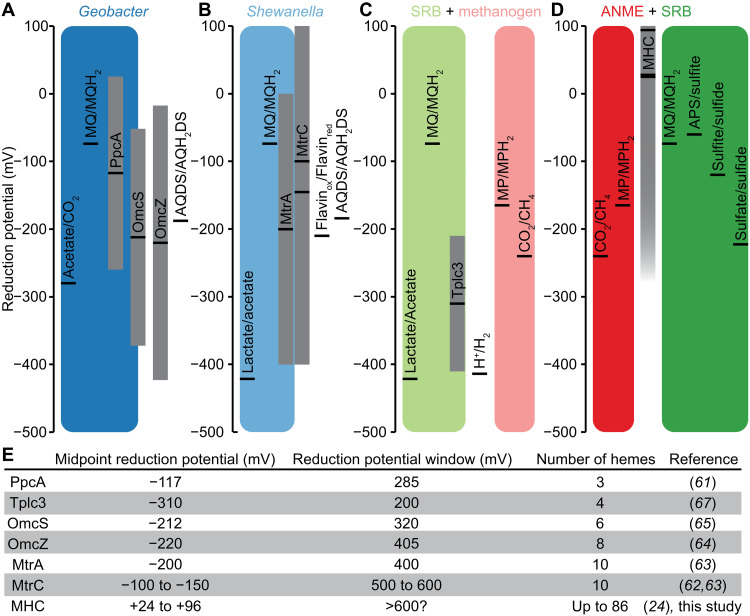
Redox properties of cytochromes in model electroactive organisms and syntrophic consortia. Electron transport routes and the reduction potentials of key components in (**A**) *Geobacter*, (**B**) *Shewanella*, (**C**) syntrophic consortia of SRB and methanogen based on interspecies hydrogen transfer, and (**D**) syntrophic consortia of ANME and SRB based on direct interspecies electron transport. Black lines indicate the midpoint reduction potentials. Gray bars indicate the reduction potential windows of multiheme cytochromes. The midpoint reduction potentials of cytochromes are more positive than their electron acceptors, but their broad redox-active windows allow reactions to occur. (**E**) Compilation of reduction potential windows show window size increase with increasing number of hemes in purified cytochromes. The large multiheme cytochrome *c* (MHC) in ANME/SRB consortia could have even broader redox-active windows to facilitate electron transport to electron acceptors at a large range of reduction potentials.

Despite the diversity of multiheme cytochrome *c* produced by ANME and SRB (*24*, *25*), we measured only a single redox potential peak at 24 to 96 mV in the three marine ANME/SRB partnerships (Fig. 2). This single peak may represent either the most abundant and redox-active cytochrome or a composite signal from multiple cytochromes with similar midpoint potentials. These values are similar to the redox potential (−20 to 100 mV) of multiheme cytochrome *c* recently identified in freshwater *Ca.* Methanoperedens, which facilitates extracellular electron transfer to insoluble electron acceptors and electrodes (*30*, *31*). However, the electron acceptors used by marine and freshwater methanotrophs differ: In marine systems, methane oxidation is coupled to sulfate reduction at relatively low potentials (−220 mV for overall sulfate to sulfide, −60 mV for adenylyl-sulfate to sulfite, and −120 mV for sulfite to sulfide) (*50*), whereas freshwater methanotrophic archaea reduce iron oxide (−100 to +100 mV) or manganese oxide (+380 mV) that are considerably more positive (*51*). Given this difference, one might expect that the cytochrome potential in ANME/SRB consortia to fall between those of methane oxidation and sulfate reduction and be more negative than the cytochromes in *Ca.* Methanoperedens, but our measured midpoint potentials are not the case (Fig. 2). This may be explained by the broad redox-active “windows” of multiheme cytochromes. In model electroactive bacteria such as *Geobacter* and *Shewanella*, cytochromes such as PpcA, OmcS, OmcZ, MtrA, and MtrC exhibit redox-active windows spanning several hundred millivolts (Fig. 4, A and B), enabling them to transfer electrons to acceptors with more negative midpoint potentials, such as anthraquinone-2,6-disulfonate (AQDS; −186 mV) and flavins (−210 mV) (*52*–*57*). These broad redox-active windows arise not only from the Nernstian behavior, where the redox potential shifts based on the reduced:oxidized ratio, but also from the overall oxidation states of the hemes and their intramolecular interactions within each cytochrome (*58*), as demonstrated in redox titration studies (*59*–*63*). A similar principle applies to syntrophy between SRB and methanogens, where interspecies hydrogen transfer is facilitated both by shifting hydrogen’s low reduction potential (−414 mV) through continuous scavenging (*64*) and by cytochrome c3 (Tplc3) having a broad 200-mV redox-active window that enables proton reduction at potentials more negative than its midpoint potential (Fig. 4C) (*65*, *66*). Our study shows ANME/SRB syntrophy operating using cytochromes with more positive midpoint potentials than the electron acceptor sulfate, possibly by a combination of high reduced:oxidized ratio and broad redox-active window (Fig. 4D). This interpretation is supported by the observation that cytochromes with more heme groups tend to exhibit even broader redox-active windows (Fig. 4E). Notably, ANME/SRB consortia harbor exceptionally large cytochromes with over 80 hemes (*15*, *24*), which likely support not only long-range electron transport but also electron acceptors with a wide range of redox potentials from metal oxides to syntrophic sulfate-reducing partners.

Overall, by measuring electron transport properties of ANME/SRB consortia, we provide direct evidence that these marine consortia use redox conduction for direct interspecies electron transport. This is much similar to electroactive *Geobacter*, *Shewanella*, and *Pseudomonas* biofilms, in which long-range electron transport occurs via multistep electron hopping through a network of bound redox cofactors such as c-type hemes in cytochromes or phenazines in extracellular DNA (*34*–*37*). Our study not only achieved highly sensitive electrochemical measurements on microliters of biomass but also confirmed the physiological relevance of these findings by measuring intact, anaerobic consortia without drying. By analyzing ANME/SRB consortia of diverse lineages, we show that these marine consortia are redox active and redox conductive, likely using their multiheme cytochrome *c* to transport electrons over distance for symbiosis. These findings deepen our understanding of microbial syntrophy and methane cycling while highlighting the broader significance of extracellular electron transfer in anoxic ecosystems.

## MATERIALS AND METHODS

### Sample collection and enrichment cultivation of ANME/SRB consortia

The ANME-1/*Desulfofervidus* (HotSeep-1) enrichment cultures originated from methane-rich, hydrothermally heated sediments of the Guaymas Basin, Gulf of California, and were cultivated at 50°C (*33*, *67*). ANME-2a/Seep-SRB1 enrichment cultures originated from cold seep sediments of Santa Monica Basin, CA, USA and were cultivated at 10°C (*32*). The ANME-2a+2c/Seep-SRB1+2 enrichment cultures originated from clastic sediments of Caldera Mud Volcano in the Mediterranean sea and were cultivated at 20°C (*33*, *67*). The seawater medium in all three types of ANME/SRB enrichments was gradually changed to the following recipe for enrichment cultivation at least 1 year before electrochemical experiments in this study to minimize mineral precipitation and avoid interference from vitamins: per liter of media in Milli-Q water (Millipore), 5.67 g of MgCl_2_·6H_2_O, 0.22 g of CaCl_2_·2H_2_O, 26.37 g of NaCl, 0.6 g of KCl, 1.5 g of Na_2_SO_4_, 0.174 g of K_2_HPO_4_, 0.107 g of NH_4_Cl, and 25 mM Hepes [4-(2-hydroxyethyl)piperazine-1-ethane-sulfonic acid] buffer at pH 7.5, with addition of trace elements solution as previously described (*17*) and without addition of vitamin solution; the pH of the medium was adjusted to 7.3, filter sterilized through a 0.22-μm filter, and sparged anoxic using N_2_ gas for at least 20 min; last, NaHCO_3_ and NaHS solutions were added from concentrated stocks to final concentrations of 5 and 0.5 mM, respectively. The community profiles of the enrichment cultures were analyzed by sequencing the V4-V5 regions of the 16*S* rRNA gene amplicons as previously described (*32*).

### Experimental treatments of ANME/SRB consortia

To investigate the electrochemical properties of ANME/SRB consortia, ANME-1/*Desulfofervidus* or ANME-2a+2c/Seep-SRB1+2 enrichment cultures were mixed, and then 2 to 4 ml of the mixture was sampled in an anaerobic chamber (N_2_ atmosphere with 2 to 4% H_2_, O_2_ < 1 part per million, humidity between 50 and 70%; Coy Laboratories, Grass Lake, MI, USA) using a 5-ml syringe with a 18-gauge needle. The subsamples were washed three times using 0.2-μm filter-sterilized anoxic artificial seawater media as above except without sulfide and trace elements, with centrifugation at 5000*g* for 1 min in between to concentrate and wash the ANME/SRB consortia.

For the heat treatment, concentrated ANME/SRB consortia were sealed in a 2-ml sterile microtube with rubber ring screw cap (SCT-200-C-S, Axygen, Union City, CA, USA) and put into a 50-ml Falcon conical centrifuge tube (Thermo Fisher Scientific, Sunnyvale, CA, USA) in the anaerobic chamber, before incubating in a high-temperature incubator at 95°C for ANME-1/*Desulfofervidus* consortia and 80°C for ANME-2a+2c/Seep-SRB1+2 consortia. For the oxygen exposure treatment, concentrated ANME/SRB consortia in 2-ml sterile microtubes (Axygen) were taken out of the anaerobic chamber and pipetted briefly to mix in ambient air, before incubating for a range of hours at room temperature.

For the paraformaldehyde treatment, 0.1 ml of N_2_-sparged anoxic 20% paraformaldehyde (Electron Microscopy Sciences, Hatfield, PA, USA) was added to 1 ml of concentrated ANME/SRB consortia and incubated at room temperature for 1 to 20 hours in the anaerobic chamber before washing three times using 1× PBS [137 mM NaCl, 2.68 mM KCl, 10 mM Na_2_HPO_4_, and 1.76 mM KH_2_PO_4_ (pH 7.4)].

### Cultivation of *G. sulfurreducens* as controls

*G. sulfurreducens* PCA (51573, American Type Culture Collection) was a gift from the laboratory stock of D. Bond at University of Minnesota (*42*). The culture was grown in 18-mm anaerobic tubes (Bellco Glass, Vineland, NJ, USA) with a black butyl rubber stopper (Geo-Microbial Technologies, Ochelata, OK, USA) at 30°C with vitamin-free anaerobic media containing 20 mM acetate as the electron donor and 40 mM fumarate as the electron acceptor and defined trace minerals (*42*) under 80% N_2_ and 20% CO_2_ atmosphere.

To obtain a *G. sulfurreducens* biofilm grown on a poised electrode, 5 ml of late-exponential culture (optical density at 600 nm = 0.45) was used to inoculate 10 ml of anaerobic media (without 30 mM fumarate) in a three-electrode electrochemical setup as previously described (*42*), with solid-state electron acceptor graphite working electrode (polished with 1500 grit, surface area of 3 cm^2^; Tri-Gemini) poised at +300 mV versus Ag/AgCl (1 M KCl) using a potentiostat (VSP300, Bio-Logic USA). A platinum wire (CHI115, CH Instruments, Austin, TX, USA) acted as the counter electrode, and a Ag/AgCl electrode in 1 M KCl solution (CHI111, CH Instruments) acted as the reference electrode. The headspace of the electrochemical reactor was constantly flushed with 80% N_2_ and 20% CO_2_ atmosphere to maintain an anoxic environment. The reactor ran for ~80 hours until the anodic current stabilized to 0.8 mA. Thereafter, a cyclic voltammogram (3 cycles at scan rate of 1 mV/s) was performed to confirm the onset potential of the catabolic current as −250 mV versus SHE. At this point, the reactor was disconnected from the potentiostat and disassembled in the anaerobic chamber. The red-colored electrode-grown biofilm, visible on the graphite electrode, was peeled off using a sterile stainless steel razor blade (AccuTec Blades, Verona, VA, USA) into a 1.5-ml sterilized microtube (MCT-150-C, Axygen) and washed three times using 1× PBS.

### Electrochemical analysis

IDA (ALS Co. Ltd., Tokyo, Japan) was used for measuring electrochemical properties of samples. The IDA microelectrodes were made of different materials—ITO (#012128), gold (#012125), or platinum (#012126)—but had the same dimensions with 65 pairs of 10-μm-wide microelectrodes separated by a 5-μm gap. Insulated wires (32 AWG, Train Control Systems, Blooming Glen, PA, USA) with exposed tips were attached to the two working electrodes on the IDA using conductive NO-VOC silver paint (SPI Supplies, West Chester, PA, USA). To protect the exposed wiring, nonconductive epoxy (Gorilla two-part clear epoxy, Cincinnati, OH, USA) was applied and allowed to cure at room temperature overnight creating a passivation layer. After preparation, the IDA was placed in a 100× 15-mm plastic petri dish (VWR International, PA, USA) for electrochemical measurements. A dual channel potentiostat (VSP200, BioLogic USA, Knoxville, TN) equipped with ultralow-current cable modules was used to perform the electrochemical measurements.

Dry conduction measurements were performed on dried biomass at room temperature in an anaerobic chamber. To decrease salt in the biomass that might contribute to conductance, the samples were washed three times using 0.2-μm-filter–sterilized anoxic deionized water with 30 s of centrifugation at 5000*g* in between before depositing onto IDA. The samples were dried onto IDA in the anaerobic chamber for 2 to 5 hours. The two working electrodes on the IDA served as the anode and cathode. For the conduction measurements, a voltage of −100 to 100 mV was applied across the anode and cathode with a scan rate of 10 mV/s. Current was monitored over the last 50% of the step duration, recorded by averaging over 50 voltage steps, and repeated one time. The resistance and conductance were calculated from the resulting ohmic current-voltage trace. The dry conductance values reported are obtained from independent biological replicates.

Redox measurements were performed on dried or wet biomass in an anaerobic chamber under conditions described above. The samples were washed three times using 0.2-μm-filter–sterilized anoxic 1× PBS before depositing onto the IDA. For drying the biomass, the samples were left in the anaerobic chamber for 2 to 5 hours. For live biomass measurements, the samples were pressed onto the IDA using Gelrite plug in a 1-ml syringe. The Gelrite (G35020-500, RPI Corp., Mount Prospect, IL, USA) was first dissolved in 1× PBS (0.5%, w/v) before solidifying in the syringe in the anaerobic chamber. After solidifying, the Gelrite plug was cut using a sterile stainless steel razor blade (AccuTec Blades) to create a flat surface. The redox measurements were performed in 1× PBS solution with a single working electrode by combining the two working electrodes of the IDA, a Ag/AgCl reference electrode (1 M KCl; CH Instruments) and a platinum wire counter electrode (CH Instruments). For cyclic voltammetry, the potential was scanned from −500 to +100 mV versus reference electrode and repeated one time, a scan rate of 10 mV/s was used, and current was measured over the last 50% of the step duration and recorded by averaging over 50 voltage steps. For the generator-collector method, one of the IDA working electrode served as the generator electrode, with the following measurement parameters: The potential was swept from +100 to −500 mV versus the reference electrode with a scan rate of 10 mV/s, and current was recorded every 0.1 s; the other IDA working electrode served as the collector electrode, holding a constant potential at +100 mV versus the reference electrode for the same duration of generator potential sweep, and current was recorded every 0.1 s. The potential values reported are obtained from independent biological replicates. All potentials in Materials and Methods are reported versus Ag/AgCl (1 M KCl) reference electrodes, as conducted in the experiments; all potentials reported in the main text, figures, and supplementary files are converted to versus SHE by adding 235 mV, corresponding to the potential difference between SHE and Ag/AgCl (1 M KCl).
