# Supplementary material for: Redox conduction facilitates direct interspecies electron transport in anaerobic methanotrophic consortia
Source: Sci Adv. 2025 Aug 22;11(34):eadw4289. doi: 10.1126/sciadv.adw4289 (PMC12372872; doi:10.1126/sciadv.adw4289)
Supplement: Supplementary file 1 — Figs. S1 to S7 [file sciadv.adw4289_sm.pdf]

Science Advances
AAAS

Supplementary Materials for

Redox conduction facilitates direct interspecies electron transport in anaerobic methanotrophic consortia

Hang Yu *et al.*

Corresponding author: Hang Yu, yuhanghank@pku.edu.cn; Victoria J. Orphan, vorphan@caltech.edu; Mohamed Y. El-Naggar, mnaggar@usc.edu

Sci. Adv. **11**, eadw4289 (2025)
DOI: 10.1126/sciadv.adw4289

This PDF file includes:

Figs. S1 to S7

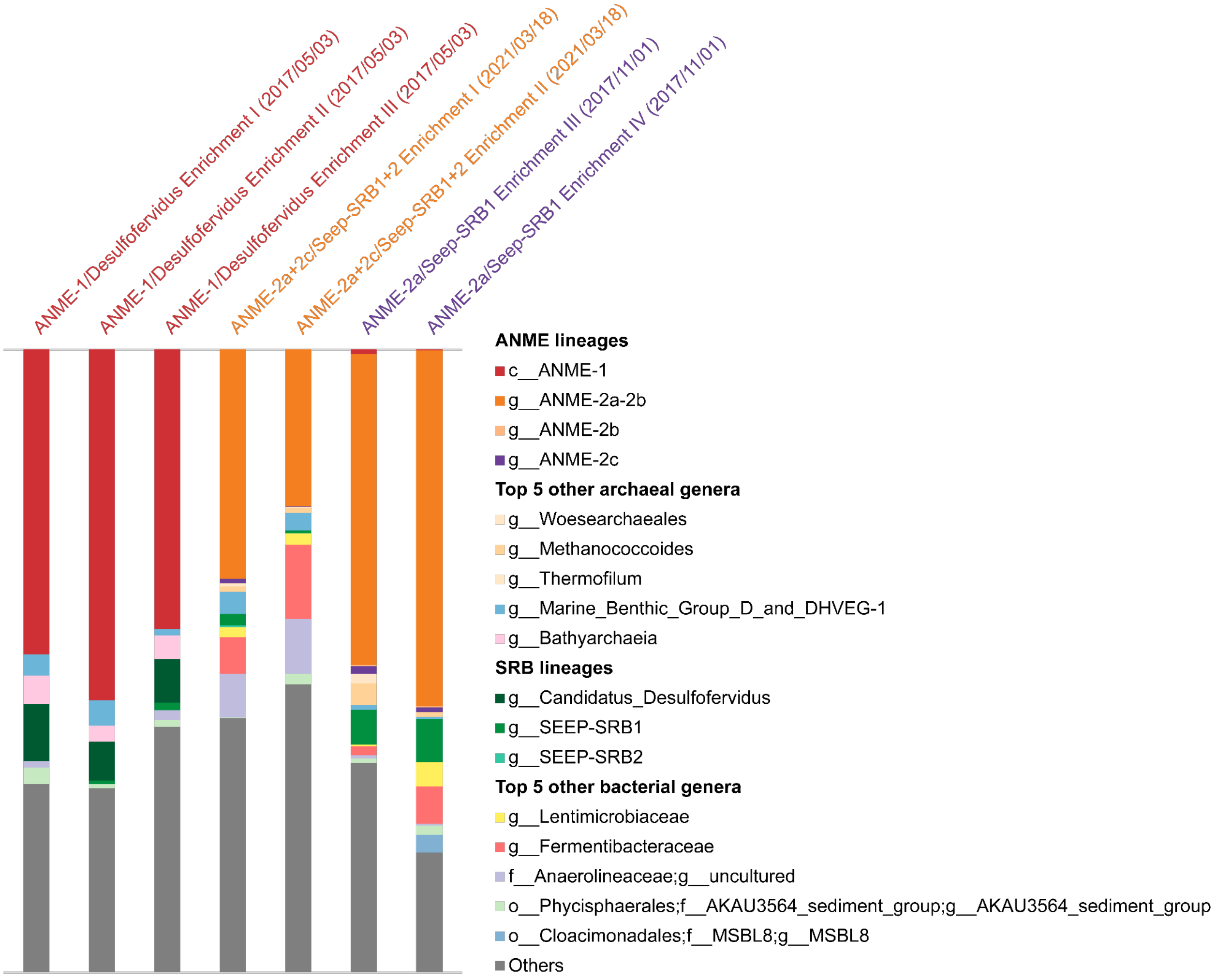

Fig. S1.

Community analysis of ANME/SRB sediment-free enrichments. V4-V5 regions of the 16S rRNA genes were PCR amplified and sequenced. Taxonomic classification is based on the SILVA SSU rRNA database v138. Data for ANME-2a/Seep-SRB1 Enrichment III/IV is from our previous publication (*32*).

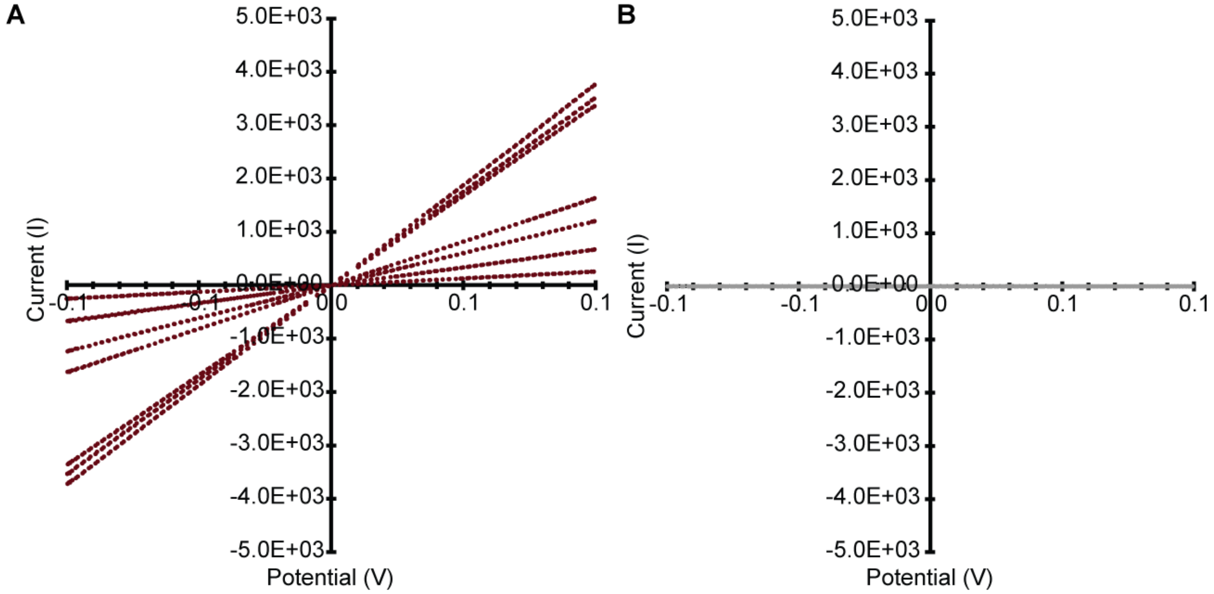

Fig. S2.

Ohmic current-voltage traces of dry conductance measurements. (**A**) Measurements of ANME-1/Desulfofervidus consortia (red) on indium tin oxide (ITO) interdigitated microelectrode arrays (IDA) show substantial dry conductance. (**B**) Measurements of blank ITO IDAs (gray) show minimal dry conductance.

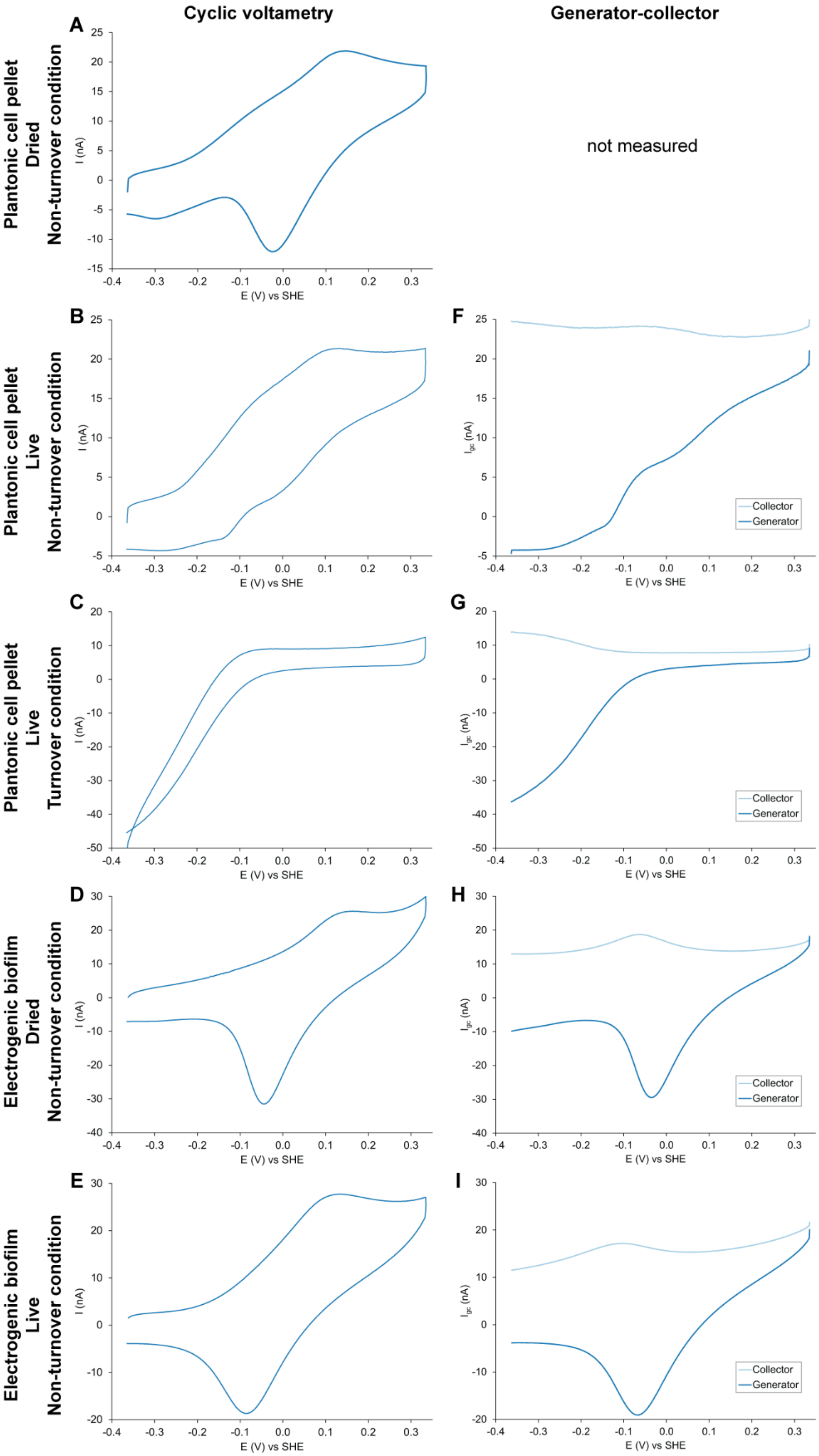
Cyclic voltametry
Generator-collector
A
B
C
D
E
F
G
H
I
Plantonic cell pellet
Dried
Non-turnover condition
Plantonic cell pellet
Live
Non-turnover condition
Plantonic cell pellet
Live
Turnover condition
Electrogenic biofilm
Dried
Non-turnover condition
Electrogenic biofilm
Live
Non-turnover condition
not measured
I (nA)
I_gc (nA)
E (V) vs SHE
Collector
Generator

Fig. S3.

Electrochemical measurements of *Geobacter sulfurreducens* PCA. Planktonic cells of *G. sulfurreducens* were grown in liquid cultures using acetate as the electron donor and fumarate as the electron acceptor. Cells were washed and pelleted, before pressing onto ITO IDAs for live electrochemistry measurements. Electrogenic biofilms of *G. sulfurreducens* were grown in a bioreactor using acetate as the electron donor and graphite electrode poised at +522 mV vs SHE as the electron acceptor. Electrogenic biofilms were washed before peeling off the graphite electrodes and pressing onto ITO IDAs for measurements. (**A**-**E**) Cyclic voltammetry measurements with scan rates of 10 mV/s. (**F**-**I**) Generator-collector measurements with generator electrodes sweeping from +335 mV to -265 mV and collector electrodes held at +335 mV.

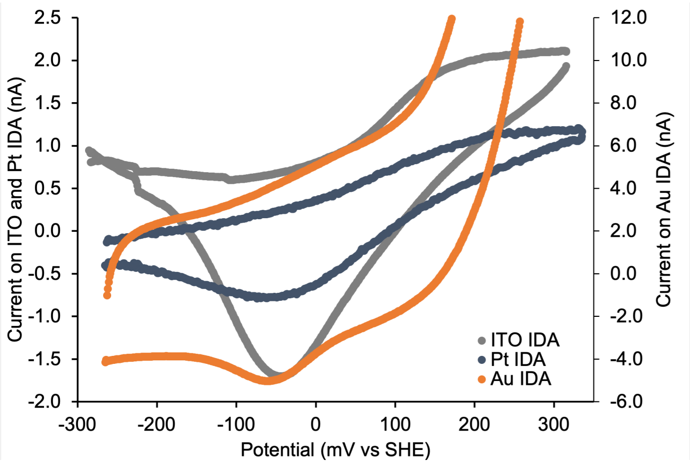

Fig. S4.

Cyclic voltammetries of ANME-1/Desulfofervidus consortia on IDAs of different electrode materials. Redox signals of different magnitudes but at similar potentials are observed from ANME/SRB consortia after background subtraction of current measured from the same blank IDA.

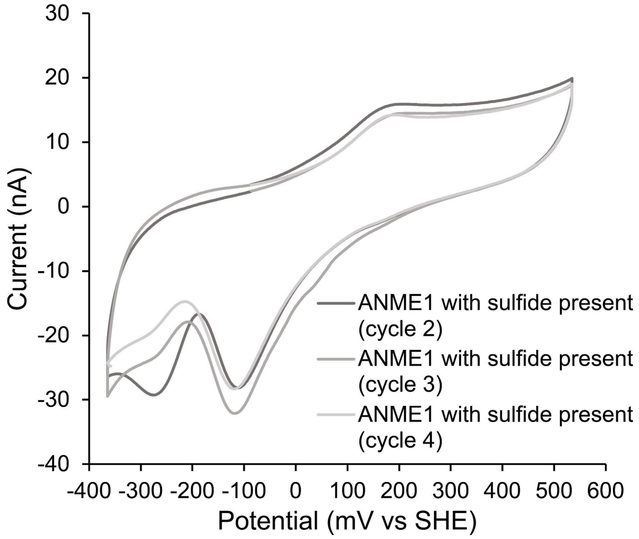

Fig. S5.

Cyclic voltammetries of ANME-1/Desulfofervidus consortia on ITO IDA with sulfide.
Besides the redox signal from the consortia, an additional cathodic peak at -275 mV that decreases with increasing scan cycles and without an anodic peak could be attributed to sulfide.

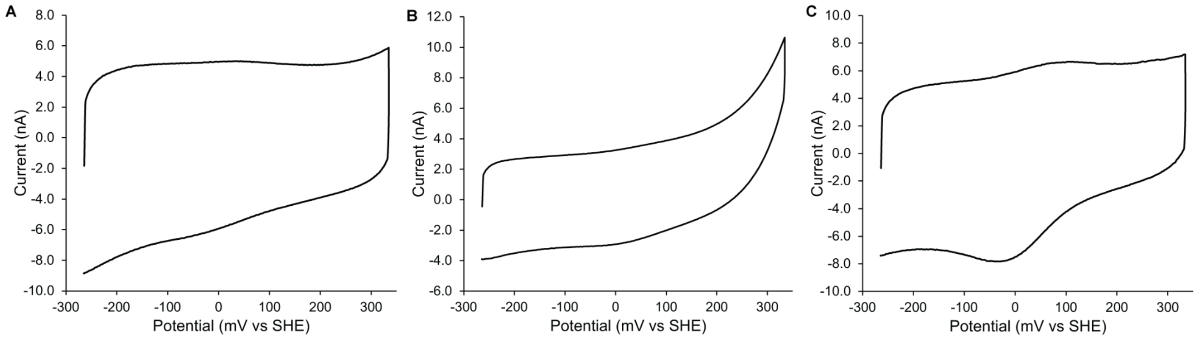

Fig. S6.

Cyclic voltammetries of dry-deposited ANME-1/Desulfofervidus consortia on ITO IDAs after treatments. (**A**) Heating consortia anoxically at 95 °C for 17 h. (**B**) Exposing consortia to oxygen for 24 h. (**C**) Fixing in paraformaldehyde for 18 h. Currents are not background subtracted. The redox signal is preserved with paraformaldehyde fixation but not heating and oxygen exposure, indicative of a biological component.

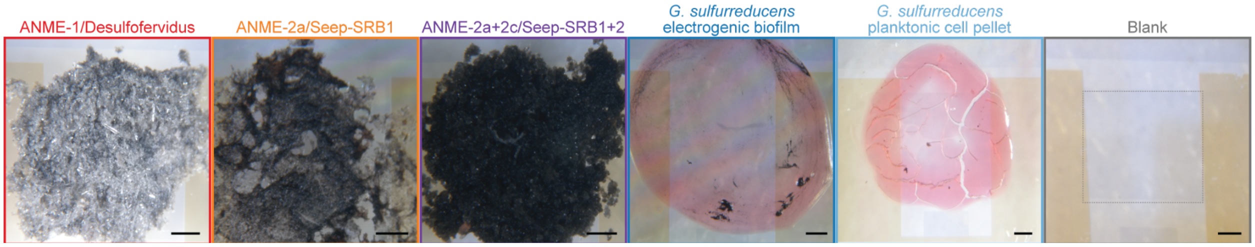

Fig. S7.

Representative light microscopy images of dried biomass on ITO IDA. In the Blank control, the dashed lined area indicates the working electrode area. Scale bars = 500 µm.
